# The Beneficial Effect of Rosmarinic Acid on Benzophenone-3-Induced Alterations in Human Skin Fibroblasts

**DOI:** 10.3390/ijms222111451

**Published:** 2021-10-23

**Authors:** Anna Galicka, Joanna Sutkowska-Skolimowska

**Affiliations:** Department of Medical Chemistry, Medical University of Bialystok, Mickiewicza 2A, 15-222 Bialystok, Poland; joanna.sutkowska@umb.edu.pl

**Keywords:** benzophenone-3, rosmarinic acid, collagen, glycosaminoglycans, decorin, elastin, MMP, hyaluronidase, elastase, fibroblasts

## Abstract

Benzophenone-3 (BP-3) is one of the most widely used chemical sunscreens. The results of many in vitro and in vivo tests confirm its high percutaneous penetration and systemic absorption, which question the safety of its wide use. The aim of our research was to assess the effect of this compound on components of the skin extracellular matrix, and to investigate whether rosmarinic acid (RA) could reduce BP-3-induced changes in human skin fibroblasts. BP-3 used at concentrations of 0.1–100 µM caused a number of unfavorable changes in the level of type I collagen, decorin, sulfated glycosaminoglycans, hyaluronic acid, elastin, and expression or activity of matrix metalloproteinases (MMP-1, MMP-2), elastase and hyaluronidase. Moreover, the intracellular retention of collagen was accompanied by changes in the expression of proteins modifying and controlling the synthesis and secretion of this protein. Most importantly, RA at a concentration of 100 µM significantly reduced or completely abolished the adverse effects of BP-3. Based on these findings, it can be concluded that this polyphenol may provide effective protection against BP-3-induced disturbances in skin cells, which may have important clinical implications.

## 1. Introduction

**Figure 1:**
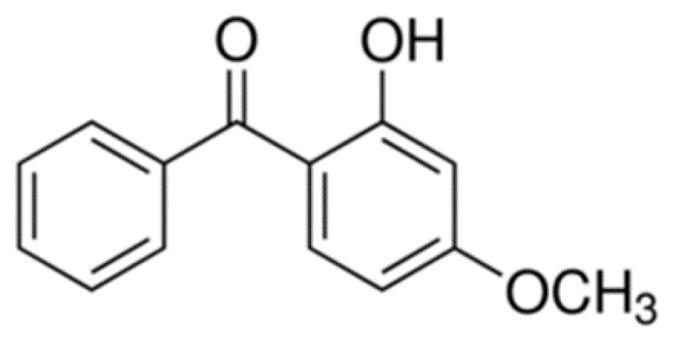
Chemical structure of benzophenone-3 (BP-3).

Ultraviolet (UV) sunscreens are compounds that have been widely used in recent years due to growing concern about the harmful effects of UV exposure associated with an increased risk of skin cancer and premature skin aging [1,2]. BP-3 (2-hydroxy-4-methoxybenzophenone, benzophenone-3, oxybenzone; CAS No. 131–57-7) (Figure 1) is one of the most widely used chemical sunscreens which is considered as a broad spectrum UV filter because it blocks both UVA and UVB radiation [3,4,5].

BP-3 as well as other chemical sunscreens such as octylmethoxycinnamate (OMC), 4-methylbenzilidenecamphor (4-MBC) and homosalate (HS) are ingredients not only of sunscreen preparations but also of many skin care and personal hygiene products for everyday use to which most people are chronically exposed. BP-3 has been approved by the Food and Drug Administration (FDA) as an ingredient in cosmetics in Europe and as an over-the-counter drug in the US. In February 2019, the US FDA issued a rule (Federal Register 84FR6204, 2019-03019) to update legal requirements for over-the-counter sales of sunscreens and to ensure their safe use [6]. BP-3 was classified in category III (those that require further testing) due to its potential photo-carcinogenic as well as estrogenic and anti-androgenic activities [5,7,8,9,10,11,12,13,14,15]. It has been reported that BP-3 at the concentration of 0.1–10 µM may increase migratory and invasive properties of both oestrogen-responsive and oestrogen-unresponsive human breast cancer cells [9,11]. Using mass spectrometry, BP-3 was detected in 83 of 120 (69%) human breast tissue samples of 40 women who have undergone a mastectomy for primary breast cancer in the range 0–26.0 ng/g of tissue [12]. Human exposure to this compound may be associated with the risk of developing endometriosis [13], Hirschsprung’s disease [14], lower testosterone levels [15], and neuronal disorders [16,17]. It is also one of the most common triggers of photoallergic skin reactions [8,18].

Production of BP-3 in Europe is as high as 100–1000 metric tons per year and continues to grow due to growing consumer demand [17]. The use of oxybenzone raises serious doubts about its benefits compared to the potentially negative health and environmental effects caused by its accumulation in the ecosystem [5,7,8,10,19]. In Hawaii, the use of BP-3 and OMC is banned since 1 January 2021 due to the harmful effects on marine ecosystems, in particular on coral reefs. The highest approved concentration of BP-3 in cosmetic sunscreen products was up to 10%, but in 2017, according to scientific reports, the European Commission reduced the use from 10% to 6% [20].

For most sunscreens, limited skin absorption has been demonstrated in a number of in vivo and in vitro tests, while BP-3 has sufficiently high penetration [5,8,21,22,23,24,25]. It has been estimated that the amount of BP-3 absorbed from the sunscreen product (a lotion containing 6% (*w*/*v*) of BP-3) over 10 h was 1–2% of the applied amount; nine healthy human volunteers were involved in this study [21]. After several applications of the recommended amount (2 mg/cm^2^) on the whole body of a sunscreen containing 4% BP-3, the mean value of this compound in the urine was higher and amounted to 3.7% [22]. Moreover, BP-3 may accumulate in the body as volunteers excrete it even five days after the last (tenth) application of the sunscreens containing this compound [22]. According to Jiang et al. [23] about 10% of a skin dose of BP-3 can be absorbed systematically. Similarly, the results obtained by Janjua et al. [24,25] confirm the percutaneous penetration of UV filters and their possible accumulation in the body. Applying 32 healthy volunteers with a sunscreen formulation containing 10% (*w*/*w*) of each BP-3, 4-MBC, and OMC to the skin, resulted in the detection of their parent forms both in plasma and in urine just 1–4 h after the whole body application (2 mg of cream per cm^2^), and the concentration was the highest for BP-3 (in plasma the increase from 3.9 ng/mL to 238 ng/mL) [25]. These results were confirmed by the latest study by Matta et al. [26] in which participants used sunscreen products containing 6% BP-3, and also from undetectable plasma levels of this compound, the geometric mean of the maximum plasma concentration increased to 209.6 ng/mL in 2 h. In an in vitro penetration study using full-thickness human skin among the five filters used, BP-3, ethylhexyl methoxycinnamate (EHM), butyl methoxydibenzoylmethane, ethylhexyl salicylate, and HS, only BP-3 and EHM (three times less than BP-3) were detected in the dermis already 30 min after applying the product [27].

Studies conducted in 2003–2004 with participants of the National Health and Nutrition Examination Survey showed the presence of BP-3 in more than 96.8% of 2517 urine samples [28]. Similarly, a Disease Control Center in the US (in 2018) reported the presence of oxybenzone in urine in about 97% of the examined 2500 individuals [8], which confirms exposure to this compound of the majority of the population. Higher levels of BP-3 were found in young girls and women than in young boys and men, which can be explained by the difference in the frequency and amount of used cosmetic products containing this compound among these groups. The detection of BP-3 in blood, plasma, and urine indicates its systemic absorption [21,22,23,24,25,26]. Oxybenzone and its metabolites have also been detected in the rat liver, kidneys, spleen, heart, brain, testes [29,30], as well as human adipose tissue [31]. Their presence in human placenta [32] and human breast milk [33] raises serious concerns about their negative impact on the development of the fetus. 

Few studies on normal human cell lines show that sunscreens, including BP-3, can induce mitochondrial stress and inhibit cell growth [34], led to deregulation of autophagy and the epigenetic state of neuronal cells, and induced their apoptosis [16,17]. However, the effects of BP-3 on human skin fibroblasts and their essential extracellular matrix (ECM) components have not been explored so far. Previously, we showed the harmful effect of parabens, also commonly used in cosmetic products, on the survival and proliferation of skin fibroblasts and metabolism of collagen type I [35,36], and most importantly, we provided evidence of the protective effect of rosmarinic acid (RA) against the negative changes exerted by methylparaben and propylparaben [36]. 

RA is an ester of caffeic acid and 3,4-dihydroxyphenyllactic acid. It has a high potential for wide introduction in pharmacy and cosmetic industries due to its strong anti-free radical activity. RA exhibits many valuable biological activities: antioxidant, anti-inflammatory, antibacterial, anti-angiogenic, antimutagenic, and antiallergic with big importance in the protection of the skin against diseases and supporting their treatment [37,38,39,40]. Furthermore, it has anti-aging properties, reduces cellulite, skin damage and accelerates wound healing [38,40]. There are many reports on the protective effect of RA or extracts rich in this compound against the harmful effects of UV radiation on the skin, which has been associated with a reduction in intracellular reactive oxygen species (ROS) [37,39,41,42,43]. In HaCaT keratinocytes, RA counteracted the oxidative stress induced by UVB by activating nuclear factor erythroid 2-related factor 2 [37]. 

In this study, we decided to assess whether BP-3 at concentrations of 0.1–100 µM might adversely affect the main structural components of the skin ECM, such as type I collagen, decorin, sulfated glycosaminoglycans (GAGs), hyaluronic acid (HA), elastin, and ECM-degrading enzymes (matrix metalloproteinases MMP-1 and MMP-2, hyaluronidase, and elastase) and if so, are they suppressed to some extent by 100 µM RA. In addition, this study took into account the effectiveness of the secretion of these macromolecules and the expression of selected factors regulating this process, mainly in the case of collagen type I. The BP-3 concentrations (0.1–100 µM) used in this study are in the same range as those used in previous cell line culture studies [11,12,16,17] and are consistent with the concentrations of this compound detected in human body (~200 µg/L in plasma [5,24,25,26] and ~5 mg/kg in adipose tissue [31]), corresponding to ~1 and ~25 µM BP-3, respectively.

## 2. Results

### 2.1. The Influence of BP-3 and BP-3 in Combination with RA on the Viability of Fibroblasts

**Figure 2:**
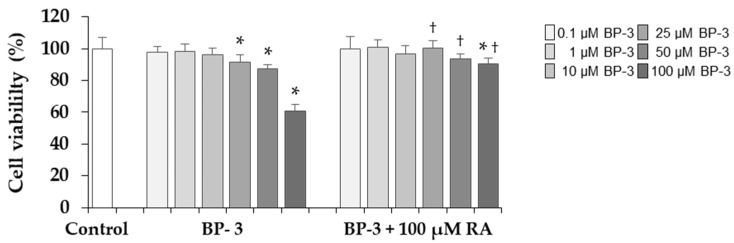
The influence of benzophenone-3 (BP-3) alone and in combination with rosmarinic acid (RA) on the viability of fibroblasts. Values represent the mean ± SD of three experiments done in duplicate; * *p* < 0.05 vs. control (untreated cells); ^†^ *p* < 0.05 vs. respective samples treated with BP-3 alone. The data are expressed as a percentage of the control sample assumed as 100%.

In order to determine the cytotoxicity of BP-3 on human skin fibroblasts, the viability of cells was determined after treatment with various concentrations (0.1, 1, 10, 25, 50, and 100 µM) of this compound for 24 h, using the MTT test. Cell viability remained unchanged in the presence of 0.1, 1, and 10 µM BP-3, while it decreased at higher concentrations (25, 50, and 100 µM BP-3) by 10%, 13%, and 40%, respectively (Figure 2). RA at the concentration of 100 µM completely prevented this decrease in cells treated with 25 and 50 µM BP-3, and partially in cells treated with the highest concentration of BP-3 (100 µM). 

### 2.2. Effect of BP-3 and BP-3 in Combination with RA on the Expression of Collagen Type I in Fibroblasts

**Figure 3:**
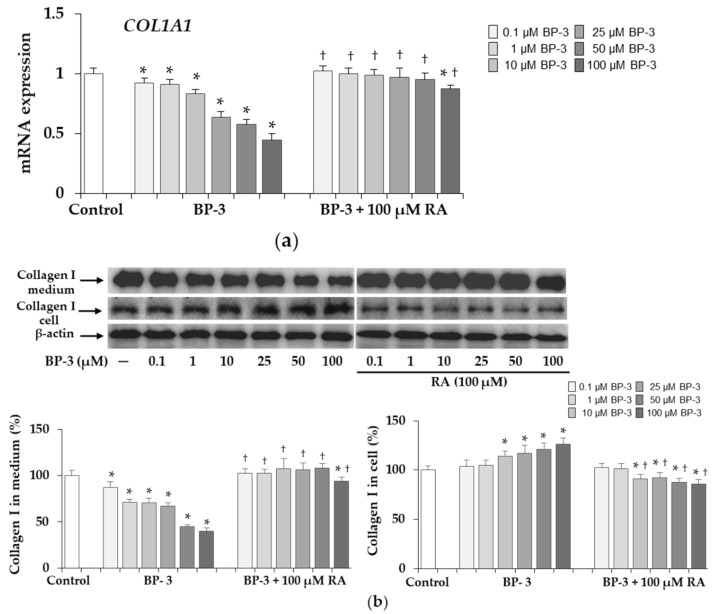
The expression of collagen type I at the mRNA (**a**) and protein (**b**) levels in fibroblasts treated with benzophenone-3 (BP-3) alone and in combination with rosmarinic acid (RA). The expression of *COL1A1* gene was assayed by real-time PCR, values represent the mean ± SD of three experiments (**a**). Representative gels of Western blotting (β-actin was used as cell protein loading control); densitometry values represent the mean ± SD of three experiments. The data are expressed as a percentage of the control sample assumed as 100% (**b**); * *p* < 0.05 vs. control (untreated cells); ^†^ *p* < 0.05 vs. respective samples treated with BP-3 alone.

The decrease in collagen expression at the mRNA level was evident at all the concentrations of BP-3 used although much more manifested at the higher concentrations (25, 50, and 100 µM), while BP-3 in combination with 100 µM RA did not affect *COL1A1* gene expression except for a slight decrease at the highest concentration of BP-3 (Figure 3a). About 90% of total type I collagen protein was identified in the control culture medium, and its significant decrease was demonstrated in the presence of BP-3, which was prevented by 100 µM RA to the same extent as at the mRNA level (Figure 3b). In turn, in cells treated with higher concentrations of BP-3 (10, 25, 50, and 100 µM) the increase in collagen type I level was observed and RA significantly reduced this effect in relation to the respective samples treated with BP-3 alone and to the control cells (Figure 3b). 

### 2.3. Expression of HSP47, Protein Disulfide Isomerase, and Glucosyltransferase in Fibroblasts under Influence of in BP-3 and BP-3 with RA

In order to explain the cause of the observed intracellular collagen type I retention under the influence of BP-3, we assessed the expression of selected molecules involved in the process of biosynthesis, stabilization and secretion of this protein. 

**Figure 4:**
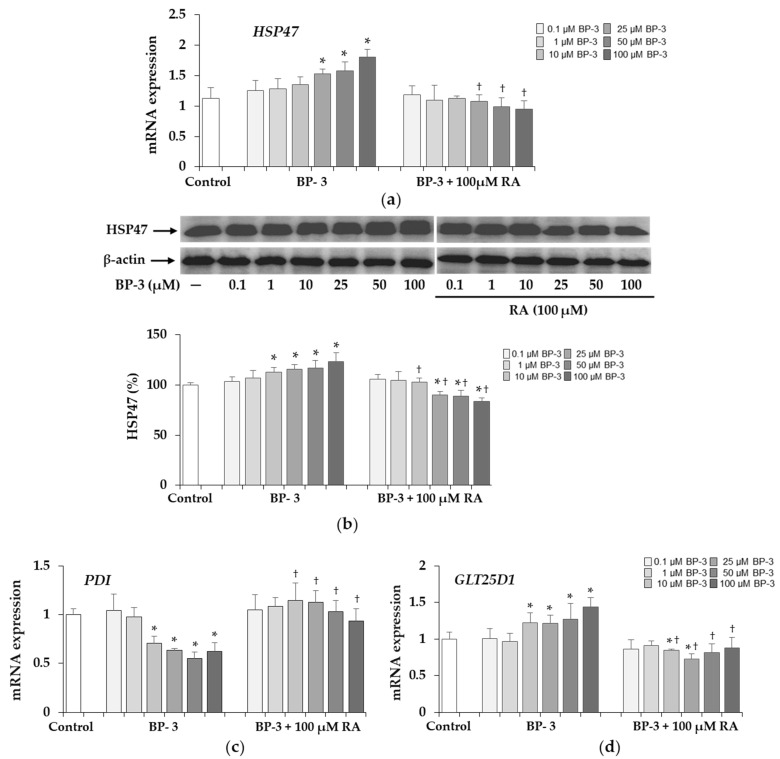
The influence of benzophenone-3 (BP-3) alone and in combination with rosmarinic acid (RA) on the expression of HSP47 at the mRNA (**a**) and protein (**b**) levels as well as on the expression of protein disulfide isomerase (*PDI*) (**c**), and glycosyltransferase 25 domains 1 (*GLT25D1*) (**d**) transcripts in fibroblasts. The expression of *HSP47*, *PDI*, and *GLT25D1* genes was assayed by real-time PCR, values represent the mean ± SD of three experiments. Representative gels of Western blotting (β-actin was used as cell protein loading control); densitometry values represent the mean ± SD of three experiments; the data are expressed as a percentage of the control sample assumed as 100% (**b**); * *p* < 0.05 vs. control (untreated cells); ^†^ *p* < 0.05 vs. respective samples treated with BP-3 alone.

The up-regulation of heat shock protein *HSP47* gene at 25, 50, and 100 µM BP-3 (Figure 4a) and HSP47 protein at 10, 25, 50, and 100 µM BP-3 (Figure 4b) was demonstrated, while adding 100 µM RA totally abolished the BP-3-induced changes. Furthermore, the protein level of this collagen was lowered not only to the respective samples treated with BP-3 alone, but also to the untreated control.

The expression of protein disulfide isomerase *(PDI*) gene decreased in the presence of 10, 25, 50, and 100 µM BP-3, but remained at the level of the control in cells exposed to the concentrations of this compound in combination with 100 µM RA (Figure 4c). The glycosyltransferase 25 domains 1 (*GLT25D1*) transcript level increased significantly in cells treated with BP-3 at concentrations of 10, 25, 50, and 100 µM, and in the presence of 100 µM RA the expression of this gene was significantly down-regulated (Figure 4d).

### 2.4. The Expression of Xbp-1 in Fibroblasts Exposed to BP-3 Alone and in Combination with RA

**Figure 5:**
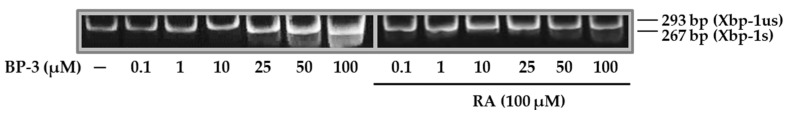
The expression of unspliced and spliced X-box binding protein 1 (*Xbp-1us* and *Xbp-1s*) in fibroblasts treated with benzophenone-3 (BP-3) alone and in combination with rosmarinic acid (RA). The PCR products were analyzed on 8% polyacrylamide gel.

To confirm whether collagen type I retention in BP-3 treated fibroblasts is related to cellular stress, we determined expression of X-box 1 binding protein (*Xbp-1s*). In cells exposed to BP-3 at concentrations of 25, 50, and 100 µM, a spliced form (*Xbp-1s*) appeared, whereas in the presence of 100 µM RA there was only the unspliced form (*Xbp-1us*) (Figure 5). 

### 2.5. Expression of Decorin in Fibroblasts Exposed to BP-3 and BP-3 in Combination with RA

**Figure 6:**
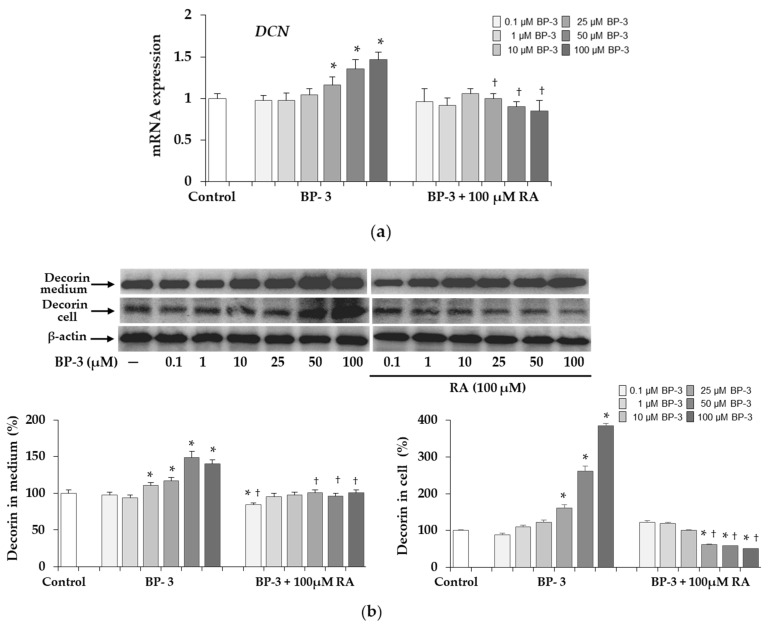
The influence of benzophenone-3 (BP-3) alone and in combination with rosmarinic acid (RA) on the expression of decorin at the mRNA (**a**) and protein (**b**) levels in fibroblasts. The expression of decorin (*DCN*) gene was assayed by real-time PCR, values represent the mean ± SD of three experiments (**a**). Representative gels of Western blotting (β-actin was used as cell protein loading control); densitometry values represent the mean ± SD of three experiments. The data are expressed as a percentage of the control sample assumed as 100% (**b**); * *p* < 0.05 vs. control (untreated cells); ^†^ *p* < 0.05 vs. respective samples treated with BP-3 alone.

Expression of the decorin (*DCN*) gene was up-regulated in the cells treated with BP-3 at concentrations of 25, 50, and 100 µM BP-3, whereas in the presence of 100 µM RA the transcript level was normalized to the control level (Figure 6a). As shown, the Western blot results (Figure 6b) BP-3 induced a significant increase in decorin expression both in cell lysates (at concentrations of 25, 50, and 100 µM BP-3) and in conditioned medium (at concentrations of 10, 25, 50, and 100 µM BP-3), with the increase in intracellular decorin levels being much greater than in the medium compared to the respective controls. In the presence of 100 µM RA there was a normalization of decorin levels and even a decrease especially in cells as compared to the control. 

### 2.6. Effect of BP-3 Alone and in Combination with RA on Sulfated GAGs Content in Fibroblasts

**Figure 7:**
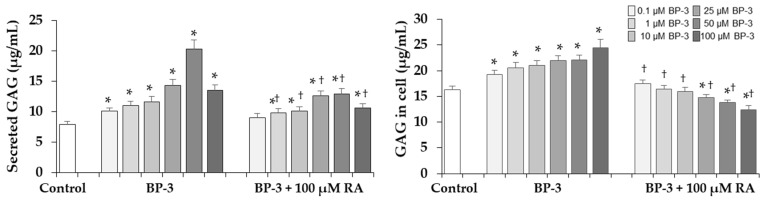
The influence of benzophenone-3 (BP-3) alone and in combination with rosmarinic acid (RA) on the content of sulfated glycosaminoglycans (GAGs) secreted into the culture medium and in cells, as determined by Glycosaminoglycan Assay Blyscan™ (Biocolor Ltd., Westbury, NY, USA). Values represent the mean ± SD of three experiments; * *p* < 0.05, vs. control (untreated cells); ^†^ *p* < 0.05, vs. respective samples treated with BP-3 alone.

In the next stage of the study, it was examined whether BP-3 also affects the sugar component of decorin, such as sulfated GAGs. As shown in Figure 7 there was a significant increase in GAG content in both the conditioned medium and in cell lysate under the influence of BP-3 at all concentrations. The beneficial effect of 100 µM RA was found because it either normalized the intracellular GAG content in cells treated with lower BP-3 concentrations or even decreased as compared to the control. In the conditioned media significant decrease in relation to the respective samples treated with BP-3 alone (except of one sample treated with 0.1 µM BP-3) was seen but their level still exceeded the control. 

### 2.7. Effect of BP-3 Alone and in Combination with RA on the Expression of HAS2, HYAL2 and HA Content in Fibroblasts

**Figure 8:**
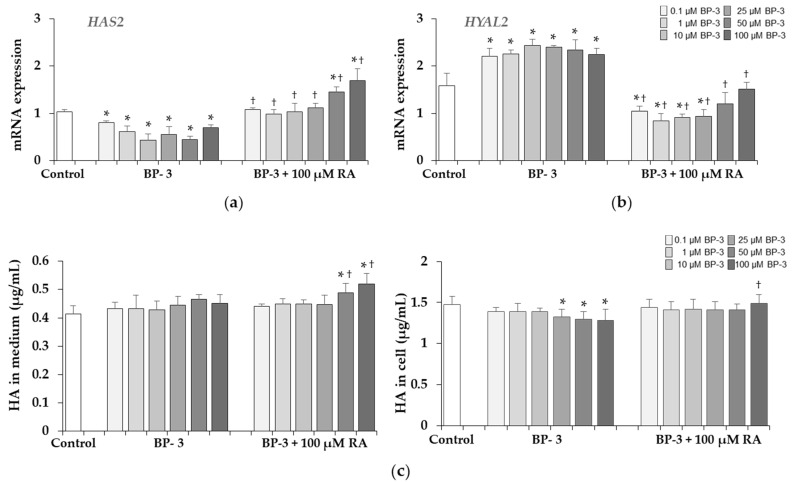
The influence of benzophenone-3 (BP-3) alone and in combination with rosmarinic acid (RA) on the expression of hyaluronan synthase 2 (*HAS2*) (**a**) and hyaluronidase 2 (*HYAL2*) (**b**) genes, and on the content of hyaluronic acid (HA) in the culture medium and cells (**c**), as determined by Hyaluronan Assay Purple-Jelley (Biocolor Ltd.). Values represent the mean ± SD of three experiments; * *p* < 0.05 vs. control (untreated cells); ^†^ *p* < 0.05 vs. respective samples treated with BP-3 alone.

The down-regulation of hyaluronan synthase 2 (*HAS2*) and up-regulation of hyaluronidase 2 (*HYAL2*) genes were found in the cells treated with BP-3 at all concentrations (Figure 8a,b). RA at the concentration of 100 µM showed the preventive effect on both genes and either prevented these changes altogether, and even increased the *HAS2* (at 50 and 100 µM BP-3) or decreased *HYAL2* (at 0.1, 1, 10, and 25 µM BP-3) expression compared to the corresponding controls. The results of HA content determination presented in Figure 8c indicated no significant effect of BP-3 on HA released into the culture medium and the inhibitory effect of higher BP-3 concentrations (25, 50, and 100 µM) on intracellular HA. RA (100 µM) increased the HA content in the conditioned medium of cells treated with 50 and 100 µM BP-3 compared to the corresponding samples treated with BP-3 alone and the untreated control as well as prevented induced by BP-3 the decrease of this GAG in cell lysate (Figure 8c). 

### 2.8. The Elastin Content and Activity of Elastase in Fibroblasts Exposed to BP-3 Alone and in Combination with RA

**Figure 9:**
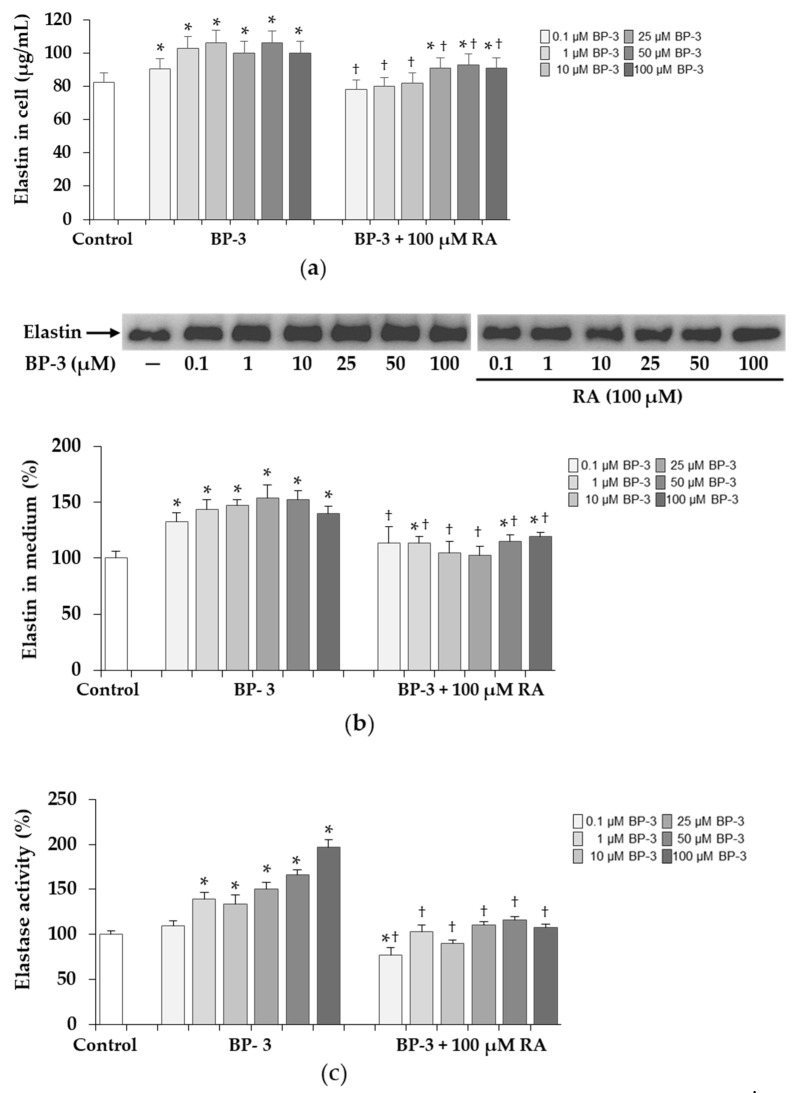
The influence of benzophenone-3 (BP-3) alone and in combination with rosmarinic acid (RA) on the content of intracellular elastin determined by Elastin Assay—Fastin™ Elastin kit (Biocolor Ltd.) (**a**) and the expression of secreted elastin determined by Western blot and densitometry (**b**), and elastase activity measured with the synthetic substrate N-succinyl-tri-alanyl-p-nitroanilide in the culture medium of fibroblasts (**c**). The data are expressed as a percentage of the control sample assumed as 100% (**b**,**c**). Values represent the mean ± SD of three experiments; * *p* < 0.05 vs. control (untreated cells); ^†^ *p* < 0.05 vs. respective samples treated with BP-3 alone.

BP-3 at all concentrations (0.1–100 µM) caused the significant increase in the content of intracellular elastin, whereas 100 µM RA entirely (at 0.1, 1, and 10 µM BP-3) or partially (at 25, 50, and 100 µM BP-3) reduced these BP-3 revealed changes (Figure 9a). The kit (Elastin Test—Fastin™ Elastin, Biocolor Ltd., Westbury, NY, USA) used in this study was only suitable for measuring elastin in cell lysates and not in conditioned media; therefore, the secreted elastin was analyzed by Western blot. As in cell lysates (Figure 9a), at all BP-3 concentrations the significant increase in elastin secreted into culture medium was seen, and in the presence of 100 µM RA, these changes were significantly reduced, and even at some BP-3 concentrations (0.1, 10, and 25 µM) they reached the control level (Figure 9b). Additionally, a test for elastase activity with a synthetic substrate was performed and its results are presented in Figure 9c. The activity was significantly upregulated by BP-3 beyond its lowest concentration and 100 µM RA completely abolished these changes. 

### 2.9. The Expression and Activity of MMP-1 and MMP-2 under Influence of BP-3 Alone and in Combination with RA in Fibroblasts

**Figure 10:**
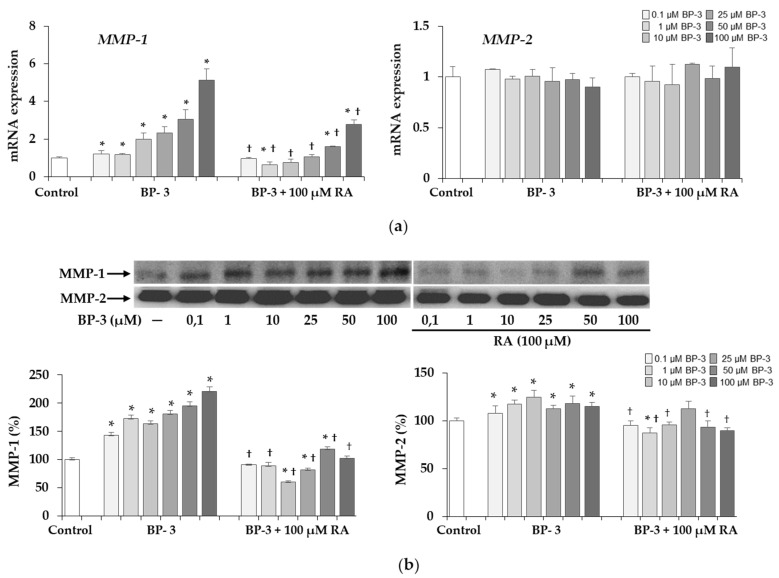

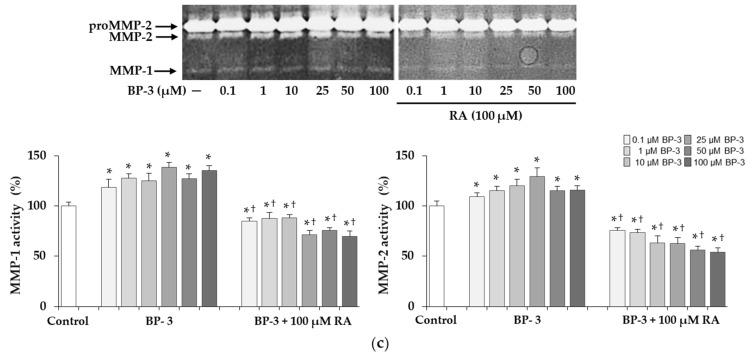
The influence of benzophenone-3 (BP-3) alone and in combination with rosmarinic acid (RA) on the expression of matrix metalloproteinases (MMP-1 and MMP-2) at the mRNA (**a**) and protein (**b**) levels and their activity (**c**) in fibroblasts. The expression of *MMP* (*MMP-1* and *MMP-2* genes was assayed by real-time PCR, values represent the mean ± SD of three experiments (**a**). Representative gels of Western blotting (**b**) and zymography (**c**); densitometry values represent the mean ± SD of three experiments. The data are expressed as a percentage of the control sample assumed as 100% (**b**,**c)**; * *p* < 0.05 vs. control (untreated cells); ^†^ *p* < 0.05 vs. respective samples treated with BP-3 alone.

The significant increases in the expression of the gene encoding MMP-1 (Figure 10a) and MMP-1 protein (Figure 10b) as well as the activity of this collagenase (Figure 10c) found with all concentrations of BP-3 were partially or completely inhibited by 100 µM RA. The inhibitory effect on MMP-1 activity demonstrated in the presence of 100 µM RA was noted not only in relation to the all corresponding samples treated with BP-3 alone, but also compared to the controls. In turn, MMP-2 expression at mRNA level was not significantly affected by either treatment with BP-3 alone or BP-3 in combination with RA (Figure 10a). However, the protein level (Figure 10b) and activity of this enzyme (Figure 10c) were higher in the culture media treated with all concentrations of BP-3 than in the control. Moreover, 100 µM RA provided (with the exception of one sample treated with 25 µM BP-3) complete protection against the stimulatory effect of this sun filter on enzyme expression and significantly reduced the activity induced by all BP-3 concentrations relative to both the corresponding samples treated with BP-3 alone and untreated controls.

## 3. Discussion

So far, no studies have been carried out on the influence of BP-3 on skin fibroblasts and on synthesized and secreted by them ECM building macromolecules. The ECM not only provides structural support to the skin but also regulates a variety of signaling pathways that govern cell proliferation, adhesion, migration, and differentiation [44]. Therefore, any changes in the biosynthesis and metabolism of individual components of the ECM may have unfavorable influence on these processes.

In this study, we showed for the first time that BP-3 as the most widely used component of sunscreen and various skin care formulations, can cause abnormalities in the biosynthesis, secretion and metabolism of collagen type I, which makes up 80–90% of total collagen and is the most abundant protein of the ECM of human skin [44,45]. Type I collagen biosynthesis is a very complex process which consists of a series of post-translational modifications, joining three procollagen chains (two α1 and one α2) and folding them into a triple right-handed helix, secretion into the extracellular space and formation of fibrils. These steps require the coordinated action of many rough *endoplasmic reticulum* (ER) molecules, including enzymes and chaperones, and the importance of these proteins in collagen biosynthesis is evidenced by many diseases, including osteogenesis imperfecta (OI) caused by their mutations [45,46,47,48]. 

BP-3 in a wide range of concentrations (from 0.1 to 100 µM) caused reduced synthesis of this key protein. The decreased protein levels resulted from down-regulated gene expression. Furthermore, intracellular retention of type I collagen was induced by BP-3 at higher concentrations (10, 25, 50, and 100 µM). Secretion of procollagen is determined by hydroxylation and glycosylation of procollagen chains and their proper folding [45,46]. The human collagen prolyl-4-hydroxylase is an enzyme composed of two α and two β subunits. The β subunit encoded by the *P4HB* gene is known as protein disulfide isomerase, which also acts as a chaperone to prevent aggregation of procollagen chains [45,47]. The significant decrease in the expression of mRNA of this protein in cells exposed to higher concentrations of BP-3 may suggest impairment of the hydroxylation process. In turn, the increase in the expression of GLT25D1 mRNA may be associated with aberrant glycosylation of procollagen chains. Collagen glycosylation takes place in the ER before triple-helix formation and is mediated by β(1-*O*)galactosyl- and α(1–2)glucosyltransferase enzymes [45,49]. Two forms of β(1-O) galactosyltransferase (GLT25D1 and GLT25D2) have been identified, while O-linked glycosylation of collagen type I is catalyzed mainly by GLT25D1 [49]. In OI, the intracellular retention is usually caused by abnormal hydroxylation and excessive glycosylation of type I collagen, which disrupts collagen secretion, and induces ER stress and subsequent the unfolded protein response (UPR) [47,48]. In our study the observed collagen retention was accompanied by the increase in the expression of the collagen chaperone HSP47 at both the mRNA and protein levels. HSP47 plays an important role in stabilizing the collagen structure, prevents the lateral connection of the triple helix during folding, and controls the secretion [50]. Furthermore, it is involved in the activation and regulation of UPR [47]. One of the UPR branch is the inositol requiring enzyme 1 (IRE1α) signaling pathway, which under homeostatic conditions is kept inactive by binding immunoglobulin protein (BiP). Under the influence of ER stress, activation of IRE1α is induced by the displacement of BiP by HSP47 accumulated in cells, which results in splicing of XBP1 mRNA [51]. The up-regulation of HSP47 and the increase in the spliced XBP1 mRNA (UPR marker) revealed in our research confirm abnormalities in the synthesis and secretion of collagen type I induced by BP-3, which could induce cellular stress.

The significant decrease in the level of collagen type I in the conditioned medium resulting from impaired secretion may have been additionally caused by increased extracellular degradation with MMP-1 and MMP-2, the induction of which was demonstrated in cells treated with BP-3. Collagenase MMP-1 specifically cleaves fibrous collagens into two separate fragments, 1/4 C-terminal and 3/4 N-terminal, whereas MMP-2 mainly known as gelatinase, can also act as collagenase, although in a weaker way [52].

After secretion, collagen type I forms fibrils and decorin is responsible for their proper structure. Decorin is the predominant proteoglycan (PG) in human dermis with a core protein of about 40 kDa and one chondroitin sulfate/dermatan sulfate GAG chain. It is distributed along collagen fibrils with the core protein and GAG chain controls the distance between the collagen fibrils [53]. By binding with collagen type I, decorin is involved in regulation of fibril diameter and orientation. The overexpression of this PG under influence of BP-3 coincided with the increase in type I collagen and HSP47 in cell lysates. It has been reported that Hsp47, which was originally characterized as the major chaperone for type I collagen, also interacts directly with decorin, possibly preventing it from binding to collagen and forming large ECM complexes inside cells [54]. However, unlike collagen, the significant increase in decorin was also found in the conditioned medium exposed to BP-3 which means that the total amount of this PG significantly exceeded the control level. Similar changes regarding reduction of type I collagen and the increase in decorin, showed by us in fibroblasts treated with the higher concentrations (50 and 100 µM) of BP-3, have been reported in other studies. Increasing expression of decorin by adenovirus in keloid fibroblasts significantly decreased collagen synthesis and stimulated the transcription level of *MMP-1* and *MMP-3* [55]. Similarly, in full-thickness punch biopsies of human skin, UV-induced decorin increase was associated with reduced expression of collagen I and III mRNA [56]. BP-3-induced alterations in collagen and decorin expression in skin fibroblasts can also be compared to the changes in human skin during aging [57,58]. 

The intracellular retention and higher level of secreted decorin protein was associated with the increased levels of sulfated GAGs both in the cell lysates and conditioned media. PGs and GAGs, although they only make up 0.2% of the dry weight of the dermis, bind large amounts of water and play a role in regulating the compressibility of the dermis [44,57]. They are responsible for moisturizing, stabilization and filling of the ECM space. HA is the only GAG that is not synthesized in the Golgi apparatus on the core protein and extruded into ECM without further modification [59]. In contrast to the significant increase in sulfated GAGs, BP-3 did not significantly affect the total content of HA, because only the decrease in its content was evident in cell lysates. HA is the main factor affecting the water content in the skin, turgidity and the diffusion gradients. Like other GAGs, it plays a space-filling and shock-absorbing role. It creates cross connections with collagen, increasing tissue stiffness. Moreover, this macromolecule is involved in various cell signaling processes [44,59]. HA also has antioxidant properties and it is believed that it can protect against the effects of reactive oxygen species (ROS) [59]. Perhaps this explains the less harmful effect of BP-3 on this component of ECM, because one of the mechanisms of the action of this UV filter is the increase in the production of free radicals and ROS [60,61]. However, it is difficult to explain and relate these much smaller changes in the amount of total HA to significant changes in the expression of enzymes mRNA involved in the synthesis (HAS2) and degradation (HYAL2) of HA. Among 3 isoenzymes HAS2 is considered the most important [62]. In our study the expression of this enzyme gene was much more decreased than the HA content under the BP-3 influence. Similarly, HAS mRNA levels in aging skin exposed to the sun were significantly reduced despite UV inducing HA synthase [58], indicating the involvement of various regulatory mechanisms at the transcription level and the protein level such as phosphorylation, glycosylation and ubiquitination [62]. Moreover, the synthesis of HA in human fibroblasts is subjected to complex regulation involving cytokines, growth factors, pH changes, and enzymatic degradation, and the mechanisms underlying them remain unclear [59,62]. Three different hyaluronidase isoforms (HYAL-1, -2 and -3) cut HA into limited fragments. In our study the significant stimulation of *HYAL2* expression by BP-3 did not reflect the amount of HA in the conditioned medium. Perhaps also in this case, mRNA expression did not correlate with enzyme activity, and if so, it is possible that the increase in hyaluronidase activity and HA degradation may stimulate the synthesis of newly synthesized HA, as was the case described by other authors [63,64]. 

The elastic fibers that make up the skin’s ECM allow it to return skin to its previous form after stretching or deformation and together with collagen it is responsible for skin firmness, elasticity, and mechanical strength. With age, the content of collagen and elastin fibers decreases, and as a consequence, the elasticity is lost and the skin wrinkles appear [58,65]. In contrast, during photoaging, there is an accumulation of disorganized elastic fibers known as “solar elastose”, but they are essentially non-functional fibers [65]. In our study, an increased content of intracellular elastin as well as secreted to the culture medium was found in the presence of BP-3. Furthermore, elastase activity increased with these BP-3-induced changes in elastin synthesis. Possibly this enzymatic increase may be the cell’s response to counteract accumulation of this protein, which requires further research and more precise quantification.

An important achievement of our research was the disclosure that RA at the concentration of 100 µM can provide complete or partial protection against these BP-3 induced adverse effects on the skin cells and the macromolecules building the skin ECM. In the previous study we proved that this polyphenolic compound itself can directly affect the transcription, increasing type I collagen or reducing collagen-degrading enzymes in both normal and OI human skin fibroblasts [36,66]. We have also given some examples of indirect protective effect of RA on collagen by influencing the factors regulating its biosynthesis, e.g., extracellular signal-regulated protein kinases 1 and 2 [36]. In the current study we also proved the beneficial effect of 100 µM RA which prevented changes induced by BP-3 in the expression of other important proteins such as chaperones (PDI, HSP47) enzymes (GLT25D1, MMP-1, MMP-2), sulfated GAGs and decorin, involved in the modification, folding, structure stabilization, secretion, fibrillogenesis, and remodelling of collagen [44,45,46,47,48,49,50]. Normalizing or significantly reducing these adverse changes in the presence of RA has prevented BP-3-induced cellular stress, which either activates UPR and partially restores cell homeostasis but may also lead to cell apoptosis [47,48,51]. 

The mechanisms of the harmful effects of UV radiation on human skin involve mainly the production of free radicals [1,2], hence the fear whether long-term use of BP-3 and other chemical filters may not protect, but intensify their formation [60,61]. It is known that ROS increase the expression of matrix metalloproteinases and serine proteases such as collagenase and elastase, thus contributing to the intensified degradation of ECM components of the skin manifested by the decrease in the skin’s resistance to stretching, elasticity and firmness, as well as skin brittleness and wrinkles [2,58]. Thus, long-term exposure of the skin to this sun filter, through the conscious and in many cases unconscious use of cosmetics containing this compound, may lead to the development of different unfavorable changes in the skin. Therefore, research aimed at looking for compounds of plant origin that protect the skin against the adverse effects of UV filters, but also show a number of other pro-health and anti-aging properties used in skin care, are of significant importance in the modern cosmetics industry. Natural compounds, such as polyphenols, carotenoids, vitamins, and anthocyanidins, which neutralize ROS resulting from the reaction of UV filters with solar radiation, may contribute to the limitation of the use of synthetic UV filters or the reduction of their harmful effect on the skin. An example is trans-resveratrol, which when applied topically inhibited lipid peroxidation and inflammation of the skin caused by UVB radiation or β-carotene, which protects the skin against stress caused by UVA radiation [67]. The combination of UV filters and antioxidants can also reduce the penetration of filters into the epidermis and dermis and improve their effectiveness through antioxidant effects. Examples of other compounds (naringenin, epigallocatechin-3-gallate in combination with hyaluronic acid, morine) acting as potential antioxidant and sunscreen agents are described in the review by Shanbhag et al. [68]. 

Likewise, RA and extracts rich in this polyphenolic compound exhibit many favorable effects against the harmful effects of UV radiation. *Rosmarinus officinalis* L. extract protected against UV-induced stimulation of MMP-1 in human skin fibroblasts and in reconstructed skin [41]. RA and *Ocimum basilicum* extract containing RA showed a protective effect on collagen and significantly improved the formation of collagen fibers in fibroblasts exposed to repeated UVA irradiation [43], while *Thunbergia laurifolia* extract rich in RA showed a protective effect on HA by inhibiting hyaluronidase activity [69]. 

The results of our current and previous studies [36] show that RA is able to protect skin cells from dysregulation of metabolism and secretion of key structural and enzymatic proteins essential for the proper functioning of ECM. We are also aware of the limitations of these studies because we have tested only one of the most common filters, but it is important to know if others also affect skin cells negatively and if so, whether RA is able to prevent these undesirable changes. Moreover, these results should be replicated on more advanced experimental models. It is worth noting, however, that our study is the first to provide important information on the potential harmful effect of BP-3 on the skin, which raises concerns about the safety of cosmetic products containing this compound and moreover, presents important practical health-promoting solution with the use of a natural polyphenolic compound.

## 4. Materials and Methods

### 4.1. Chemicals

RA was a product of BIOKOM (Warsaw, Poland). BP-3, radioimmunoprecipitation assay (RIPA) buffer, [3-(4,5-dimethylthiazol-2-yl)-2,5-diphenyltetrazolium bromide] (MTT), dimethyl sulfoxide (DMSO), sodium dodecyl sulfate (SDS), bovine serum albumin (BSA), gelatin, acrylamide and N,N’-Methylenebisacrylamide, protease inhibitor cocktail (P8340), and N-succinyl-tri-alanyl-p-nitroanilide (STANA) were provided by Sigma-Aldrich Corp. (St. Louis, MO, USA). Dulbecco’s minimal essential medium (DMEM), phosphate-buffered saline (PBS), and fetal bovine serum (FBS) used in the cell culture were purchased from Gibco (Thermo Fisher Scientific, Waltham, MA, USA). Penicillin, streptomycin, and glutamine were obtained from Quality Biologicals Inc. (Gaithersburg, MD, USA). 

### 4.2. Fibroblast Culture and Treatment

The study was performed on the normal human skin fibroblast line (CRL-1474) purchased from American Type Culture Collection (Manassas, VA, USA). Fibroblasts were cultured in DMEM (Gibco, Thermo Fisher Scientific, Waltham, MA, USA) supplemented with 10% FBS (Gibco, Thermo Fisher Scientific, Waltham, MA, USA) as well as 2 mM glutamine (Quality Biologicals Inc., Gaithersburg, MD, USA), penicillin (50 U/mL) (Quality Biologicals Inc., Gaithersburg, MD, USA), and streptomycin (50 µg/mL) (Quality Biologicals Inc., Gaithersburg, MD, USA) at 37 °C in a humidified incubator in atmosphere containing 5% CO_2_. For experiments fibroblasts were grown to 90% confluence and the cultured medium was changed to DMEM (Gibco, Thermo Fisher Scientific, Waltham, MA, USA) without serum before addition of compounds. BP-3 (Sigma-Aldrich Corp., St. Louis, MO, USA) and RA (BIOKOM, Warsaw, Poland) were dissolved in DMSO (Sigma-Aldrich Corp., St. Louis, MO, USA) and stored as the concentrated solutions at 4 °C. Fresh dilutions using DMEM (Gibco, Thermo Fisher Scientific, Waltham, MA, USA) were prepared just before adding the reagents to the cells with the final DMSO (Sigma-Aldrich Corp., St. Louis, MO, USA) concentration not exceeding 0.1% (*v*/*v*). 

### 4.3. Determination of Cell Viability with MTT Test

Fibroblasts were cultured in 96-well plates (1 × 10^4^ cells per well) for 24 h. The compounds were added for 24 h and after this time the culture medium was removed and cells were washed three times with PBS (Gibco (Thermo Fisher Scientific, Waltham, MA, USA). MTT (Sigma-Aldrich Corp., St. Louis, MO, USA) solution (0.5 mg/mL) was added and cells were incubated for 4 h at 37 °C. After this time MTT solution was removed and 100 μL of DMSO (Sigma-Aldrich Corp., St. Louis, MO, USA) and 12.5 μL of Sorensen’s glycine buffer was added to dissolve formazan crystals on a plate shaker. Cell viability was evaluated by the measurements of the absorbance at 570 nm using a microplate reader (TECAN, Männedorf, Switzerland).

### 4.4. Quantitative Real-Time PCR Analysis

Fibroblasts were seeded at a density of 1 × 10^5^ cells per well in 6-well plates. After 24 or 48 h incubation in DMEM (Gibco, Thermo Fisher Scientific, Waltham, MA, USA) with 10% FBS (Gibco, Thermo Fisher Scientific, Waltham, MA, USA), the medium was replaced with a new serum-free one and cells were treated with studied compounds for 24 h. After that time the conditioned media were collected and stored at −20 °C, whereas cells after washing with PBS (Gibco, Thermo Fisher Scientific, Waltham, MA, USA) were harvested for RNA isolation using a Total RNA Mini Plus concentrator (A&A Biotechnology, Gdynia, Poland). The concentration of isolated RNA was measured using NanoDrop 2000 spectrophotometer (Thermo Fisher Scientific, Waltham, MA, USA) and the equal amounts (1 µg) were used to the synthesis of complementary DNA (cDNA) with the cDNA Synthesis Kit (Bioline, London, UK). For quantitative Real-time PCR (qRT-PCR) cDNA samples were diluted 10-fold and the reactions were performed in the CFX96 Real-Time System thermal cycler (Bio-Rad, Hercules, CA, USA) using the SensiFAST™ SYBR kit (Bioline, London, UK). The sequences of primers (Genomed, Warsaw, Poland) used in the reactions are given in the Table 1. The qRT-PCR parameters were as follows: 30 s at 95 °C for initial denaturation, followed by 40 cycles: 10 s at 95 °C (denaturation), 10 s at 60–62 °C (annealing), and 20 s at 72 °C (extension). The reaction products were verified by analysis of melting curves. The 2^−ΔΔCT^ method in the CFX96 Real-Time PCR System (Bio-Rad, Hercules, CA, USA) was used to assess the relative level of gene expression.

### 4.5. XBP1 Splicing Analysis

Equal amounts of the isolated RNA (1 µg) were used for PCR amplification and the 0.3 μM each primer: sense (5′-TCAGCTTTTACGAGAGAAAACTCATGGCCT-3′) and antisense (5′-AGAACATGTGTGTCGTCCAAGTGTGTCGTCCAAGTGTG) purchased in Genomed (Warsaw, Poland). Samples were incubated 30 min at 50 °C and then reactions were repeated 30 times at 94 °C, 60 °C, and 72 °C for 30 s at each temperature in the CFX96 Real-Time System thermal cycler (Bio-Rad, Hercules, CA, USA). Reaction products were analyzed by electrophoresis on 8% acrylamide gel and visualized by ethidium bromide staining.

### 4.6. Western Blot

The conditioned media were concentrated 10 times using Centrifugal Filter Units (10K) (Merck Millipore Ltd., Carrigtwohill, County Cork, Ireland) and the concentration of total protein was measured using Coomassie Plus—The Better Bradford Assay Reagent (ThermoFisher Scientific, Rockford, IL, USA). Cell layers were harvested in RIPA buffer (Sigma-Aldrich Corp., St. Louis, MO, USA) supplemented with protease inhibitor cocktail (P8340) (Sigma-Aldrich Corp., St. Louis, MO, USA). After incubation on ice for 20 min and centrifugation (18,000× *g* for 20 min at 4 °C), the total protein concentration in cell lysates was determined using the BCA Protein Assay Kit (Pierce, Rockford, IL, USA). For Western blot analysis, an equal amount of protein (20 µg) was loaded on 7.5% or 10% polyacrylamide gels (depending on the molecular mass of analyzed proteins) and after electrophoresis proteins were transferred onto Immobilon-P Transfer membranes (Merck Millipore Ltd., Tullagreen, Carrigtwohill, County Cork, Ireland). Membranes were blocked with 5% (*w*/*v*) non-fat dried milk in 50 mM Tris-HCl, pH 7.5, 500 mM NaCl, 0.05% (*v*/*v*) Tween 20 solution (TBS-T) for 1 h at room temperature. After washing with TBS-T, membranes were incubated overnight at 4 °C with primary antibody solution: monoclonal antibodies against collagen type I, decorin, elastin, HSP-47, MMP-1 and MMP-2 (1:1000; Santa Cruz Biotechnology Inc., Santa Cruz, CA, USA) and monoclonal antibody against β-actin (1:1000; Sigma-Aldrich Corp., St. Louis, MO, USA). In order to analyze these proteins, peroxidase-conjugated anti-mouse immunoglobulin G (IgG) (whole molecule) (Sigma-Aldrich Corp., St. Louis, MO, USA) as a secondary antibody at the concentration of 1:2000 (in TBS-T containing 5% dried milk) was added. The membranes were incubated for 1 h under gentle shaking, rinsed with TBS-T (5 times for 5 min), and finally subjected to Westar Supernova Chemiluminescent Substrate for Western Blotting (Cyanagen, Bologna, Italy). The intensity of the protein bands was measured by densitometry using an imaging densitometer (G:BOX, Syngene, Cambridge, UK) and normalized to the corresponding β-actin as a loading control.

### 4.7. The Quantitative Measurement of Total Sulfated Glycosaminoglycans, Hyaluronic Acid, and Elastin

The amount of total sulfated glycosaminoglycans, hyaluronic acid, and elastin was measured separately in cell lysates and conditioned media using Glycosaminoglycan Assay Blyscan™, Hyaluronan Assay Purple-Jelley, and Elastin Assay—Fastin™ Elastin, respectively; all kits were from the company Biocolor Ltd. (Westbury, NY, USA). Conditioned media were 10-times concentrated (Centrifugal Filter Units (10K)), Merck Millipore Ltd., Carrigtwohill, County Cork, Ireland) prior to the quantification; all measurements were made according to the manufacturers’ recommendations. 

### 4.8. The Measurement of Elastase Activity

Elastase activity in the conditioned medium of skin fibroblasts was measured using the synthetic substrate N-succinyl-tri-alanyl-p-nitroanilide (STANA) (Sigma-Aldrich Corp., St. Louis, MO, USA). Briefly, 2 µL of 62.5 mM STANA was mixed with 100 µL of the conditioned medium and incubated for 1 h at 37 °C. The release of p-nitroaniline was determined by measuring absorbance at 410 nm using a microplate reader (TECAN, Männedorf, Switzerland). Enzymatic activity was expressed as unit/mg of protein, where 1 unit corresponds to the activity that releases 1 nmol of nitroaniline per 1 h.

### 4.9. Zymography

The conditioned media containing an equal amount of total proteins were mixed with 4 times concentrated sample buffer and separated by non-reducing sodium dodecyl sulfate polyacrylamide gel electrophoresis (SDS–PAGE). The gelatin (1 mg/mL) (Sigma-Aldrich Corp., St. Louis, MO, USA) was added during preparation of 10% polyacrylamide gel. After electrophoresis, gels were incubated for 30 min at room temperature with gentle shaking in 2.5% Triton X-100 (Sigma-Aldrich Corp., St. Louis, MO, USA) solution in order to remove SDS (Sigma-Aldrich Corp., St. Louis, MO, USA). The renaturing buffer was replaced with developing buffer (50 mM Tris-HCl, pH 8.0, 5 mM CaCl_2_, 5 µM ZnCl_2_, and 0.02% NaN_3_) and gels were incubated overnight at 37 °C with gentle shaking. Finally, they were stained with Commassie blue R-250 (Sigma-Aldrich Corp., St. Louis, MO, USA) staining solution for at least 30 min until the gel was uniformly dark blue, and then destained until areas of enzyme activity appeared as clear bands against dark blue background. Images of the zymograms were subjected to the densitometric analysis using G:BOX, Syngene (Cambridge, UK).

### 4.10. Statistical Analysis

The results were analyzed using the Statistica 12 software (StatSoft, Tulsa, OK, USA). They were presented as the mean ± standard deviation (SD). Statistical differences were estimated using a one-way ANOVA followed by Tukey’s test, and values of *p* < 0.05 were considered as statistically significant.

## 5. Conclusions

This study provides new insight into the effect of BP-3 on human skin cells and on the macromolecules they synthesize (type I collagen, decorin, sulfated GAG, HA, and elastin), which make up the main part of the skin tissue, the extracellular matrix. These macromolecular components form complex networks, which are involved in interactions with cells by binding to cell surface receptors and regulate cell phenotype and function to maintain homeostasis of the skin tissue. Our research for the first time revealed changes in synthesis, secretion and degradation of these ECM components in BP-3 exposed skin fibroblasts. Moreover, the intracellular retention of type I collagen along with decorin, associated with the increase in the level of the chaperone HSP47 and the splicing form of Xbp-1s indicated the induction of cellular stress. If the homeostasis is not restored, it may contribute to the deterioration of cell functioning and their increased apoptosis, which is a characteristic feature of the aging process.

A significant achievement of our research is the disclosure that rosmarinic acid can largely or even completely prevent most of these adverse changes caused by BP-3 in skin fibroblasts. It should be especially emphasized that the protective properties of RA against the harmful effects of BP-3 and parabens, demonstrated in our current and previous studies, significantly increase the therapeutic potential of this natural compound and its practical implications in the cosmetics industry. Since RA itself is an effective antioxidant that protect against UV rays, if it does not replace the synthetic UV filters, it can reduce their unfavorable effects on human health.

## Figures and Tables

**Table 1:** Sequences of primer used in the quantitative Real-Time PCR.

Gene	Primer Sequence	
*COL1A1*	forward	5′-GCTCGTGGAAATGATGGTGC-3′
	reverse	5′-ACCCTGGGGACCTTCAGAG-3′
*MMP-1*	forward	5′-CATTGATGGCATCCAAGCC-3′
	reverse	5′-GGCTGGACAGGATTTTGGG-3′
*MMP-2*	forward	5′-TGTGTCTTCCCCTTCACTTT-3′
	reverse	5′-GATCTGAGCGATGCCATCAA-3′
*DCN*	forward	5′-TGATGCAGCTAGCCTGAAAGG-3′
	reverse	5′-AGGCGTGTTGGCCAGAGAG-3′
*HSP47*	forward	5′-AGAGGTCACCAAGGATGTGGAG-3′
	reverse	5′-TGGGGCATGAGGATGATGAG-3′

## Abbreviations

BP-3	Benzophenone-3
BiP	Binding immunoglobulin protein
*DCN*	Gene coding decorin
ECM	Extracellular matrix
EHM	Ethylhexyl methoxycinnamate
ER	Endoplasmic reticulum
GAG	Glycosaminoglycan
GAPDH	Glyceraldehyde-3-phosphate dehydrogenase
GLT25D1	Collagen beta(1-O)galactosyltransferase 1
HA	Hyaluronic acid
HAS-2	Hyaluronan Synthase 2
HS	Homosalate
HSP47	Heat Shock Protein 47
HYAL-2	Hyaluronidase 2
IRE1α	Inositol requiring enzyme 1
4-MBC	4-methylbenzilidenecamphor
MMP	Matrix metalloproteinase
OI	Osteogenesis imperfecta
OMC	Octylmethoxycinnamate
P4HB	Prolyl 4-hydroxylase subunit beta
PDI	Protein disulfide isomerase
PG	Proteoglycan
RA	Rosmarinic acid
ROS	Reactive oxygen species
STANA	N-succinyl-tri-alanyl-p-nitroanilide
UPR	Unfolded protein response
UV	Ultraviolet
Xbp-1s	X-box 1 binding protein
